# Risk Stratification of Sudden Cardiac Death in Patients with Heart Failure: An update

**DOI:** 10.3390/jcm7110436

**Published:** 2018-11-10

**Authors:** Daniele Masarone, Giuseppe Limongelli, Ernesto Ammendola, Marina Verrengia, Rita Gravino, Giuseppe Pacileo

**Affiliations:** 1 Heart Failure Unit, AORN dei Colli, Monaldi Hospital, 80121 Naples, Italy; limongelligiuseppe@libero.it (G.L.); ammendolaernesto@libero.it (E.A.); mariverr@yahoo.it (M.V.); ritagravino@virgilio.it (R.G.); gpacileo58@tin.it (G.P.); 2 Department of Translational Medical Sciences, Luigi Vanvitelli University, 80121 Naples, Italy; 3 Institute of Cardiovascular Sciences, University College of London, London WC1E 6BT, UK

**Keywords:** sudden cardiac death, heart failure reduced ejection fraction, heart failure preserved ejection fraction

## Abstract

Heart failure (HF) is a complex clinical syndrome in which structural/functional myocardial abnormalities result in symptoms and signs of hypoperfusion and/or pulmonary or systemic congestion at rest or during exercise. More than 80% of deaths in patients with HF recognize a cardiovascular cause, with most being either sudden cardiac death (SCD) or death caused by progressive pump failure. Risk stratification of SCD in patients with HF and preserved (HFpEF) or reduced ejection fraction (HFrEF) represents a clinical challenge. This review will give an update of current strategies for SCD risk stratification in both HFrEF and HFpEF.

## 1. Introduction

Heart failure (HF) is a complex clinical syndrome in which structural/functional myocardial abnormalities result in symptoms and signs of hypoperfusion and/or pulmonary or systemic congestion at rest or during exercise [1].

Currently, 5.7 million people in the United States have HF, and approximately 45% of them have systolic dysfunction (reduced ejection fraction) [2]. By 2030, it is expected that more than eight million people in the United States will have this condition [3]. In Europe, the EPICA (EPidemiologia da Insuficiencia Cardiaca e Aprendizagem) study, enrolled 5434 subjects with an age of >25 years, and reported an average HF prevalence in the general population of 9.8%, with an incidence rate that increased with age [4]. More than 80% of deaths in patients with HF recognize a cardiovascular cause, with most being either sudden cardiac death (SCD) or death caused by progressive pump failure [5].

SCD is defined as death from unexpected circulatory arrest occurring within an hour of the onset of symptoms or during sleep [6]. In most cases, SCD is triggered by an arrhythmic event (i.e., ventricular tachycardia, ventricular fibrillation), although pulseless electrical activity has been recently reported as a frequent cause of SCD [7].

Risk stratification of SCD in patients with HF reduced ejection fraction (HFrEF) represents a clinical challenge. Current guidelines proposed an algorithm based on left ventricular ejection fraction (LVEF) that is considered the only parameter to identify high-risk patients [8]. However, it is widely known, that the current approach is not able to stratify population and the spectrum of risk with a high level of accuracy.

For this reason, a multiparametric approach is needed for more precise detection of HFrEF patients that can receive benefit from an implantable cardioverter defibrillator (ICD) implantation. HF, with preserved ejection fraction (HFpEF), represents about 50 % of HF patients in a recent series [9], although SCD has been estimated to be a frequent cause of death in HFpEF (about a quarter of all deaths), a specific strategy for identifying high-risk patients is unknown.

This review will give an update of current strategies for SCD risk stratification in both HFrEF and HFpEF.

## 2. Research Strategy

By using the terms sudden cardiac death, heart failure reduced ejection fraction, heart failure reduced ejection fraction implantable cardioverter defibrillator, and implanted cardioverter-defibrillator, we searched the MEDLINE database and the Cochrane Central Register of Controlled Trials without year or language restrictions. We also searched the ClinicalTrials.gov and Current Controlled Trials (controlled-trials.com) registries for any studies not yet published in journals.

## 3. Epidemiology of SCD

Before disease-modifying therapies became available, the incidence of SCD in patients with HFrEF was higher than 20% per year [10], nevertheless with current pharmacologic and electric therapy, the incidence of SCD has decreased to about 3% per year [11].

Currently, SCD accounts for about 40% to 45% of all deaths in HFrEF patients, and the proportion of SCD is higher in patients with milder symptoms (New York Heart Association (NYHA) class II-III) [12], indeed two-thirds of patients with NYHA functional class II, experience SCD, compared with only one-third of those with NYHA functional class IV symptoms, who died preponderantly for advanced HF [13].

Limited epidemiological data are available exploring the landscape of SCD in patients with HFpEF, however recent trials reported that SCD occurs in about 20% of patients with HFpEF [14].

## 4. Pathophysiology of SCD

**Figure 1:**
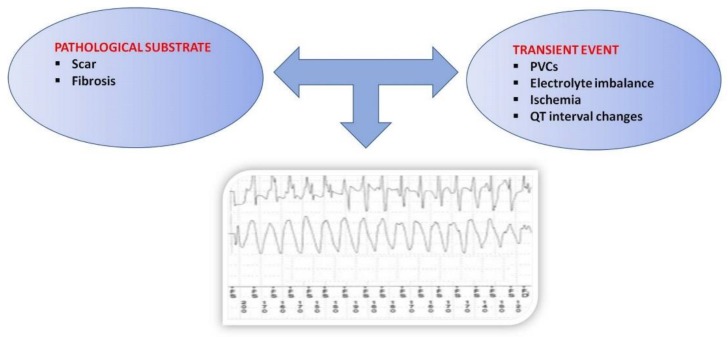
Anatomic substrate and pathophysiology of sudden cardiac death in heart failure reduced ejection fraction. PVCs: Premature ventricular complex; QT: QT interval.

The mechanisms of SCD in patients with HFrEF are complex and require the chance interaction between a transient event and underlying pathologic substrate that induces electrical instability [15] (Figure 1).

In ischemic cardiomyopathy, SCD is related to areas of the prior infarcted myocardium that are adjacent to thick scar that has formed over time. Residual endomyocardial fibers survive because blood nourishes them within the left ventricular cavity (i.e., retrograde perfusion through the left atrial venous system, retrograde perfusion through sinusoidal channels, or simply by diffusion of oxygen from left ventricular cavity blood flow through the endocardium) [16]. These surviving myocytes become embedded within regions of fibrosis that constitute the substrate for abnormal non-uniform anisotropy with a conduction block and propagation barrier that promotes reentry and consequently ventricular arrhythmias [17,18]. In patients with systolic dysfunction after a myocardial infarction, non-arrhythmic SCD frequently occurs during the first four to six weeks and is due to mechanical complication of myocardial infarction (i.e., rupture of the left ventricular free wall; rupture of the interventricular septum; and the development of acute mitral regurgitation) [19]. It seems that the proportions of arrhythmic and non-arrhythmic SCD cases become equivalent approximately one month after the acute coronary syndrome. These observations explain the guidelines recommendation of delaying ICD implantation for 40 days after myocardial infarction [20,21]. In non-ischemic cardiomyopathy, ventricular myocardium has multiple patchy areas of fibrosis without significantly visible scar [22]. This finding explains why reentry accounts for only 40% of the mechanisms of ventricular arrhythmias in patients with non-ischemic cardiomyopathy, with the rest due to triggered activity (i.e., early afterdepolarizations and delayed after depolarizations) [23,24].

The primary mechanism of SCD in patients with HFpEF seem related to myocardial fibrosis that alters regional conduction patterns and serves as islands of reentry [25]. Furthermore, ischemia is likely an under-recognized contributor to ventricular arrhythmias in HFpEF [26].

Irrespective of HF etiology and LVEF, in patients with advanced HF, arrhythmias are triggered primarily by pump failure with about 60% of such patients that have severe bradyarrhythmias or electromechanical dissociation as the underlying cause for their SCD [27].

## 5. Risk Stratification of SCD in HFrEF

Even though several risk factors for SCD have been identified and proposed in HFrEF patients (Table 1), risk stratification to identify those who will benefit in primary prevention of ICD implantation remains a challenge.

## 6. Cardiac Imaging

In the recent ESC Guidelines for HF and SCD, LVEF is widely used to identify candidates for primary prevention therapy with ICD, but this approach has significant and well-recognized limitations [28,29]. Importantly, contemporary real-world data indicate that annually no more than 3% to 5% of ICD implanted for primary prevention deliver life-saving therapies [30,31]. Furthermore, in the Oregon Sudden Unexpected Death Study (a community-based study with an enrolled population of one million patients), of 2093 patients with SCD, only 20.5% had LVEF ≤35% [32]. For this reason, new markers that will enhance SCD risk stratification are needed. Recently, a novel non-invasive imaging technique has been proven to add incremental value over LVEF to identify patients who may benefit from an ICD implantation.

### 6.1. Speckle Echocardiography 

**Figure 2:**
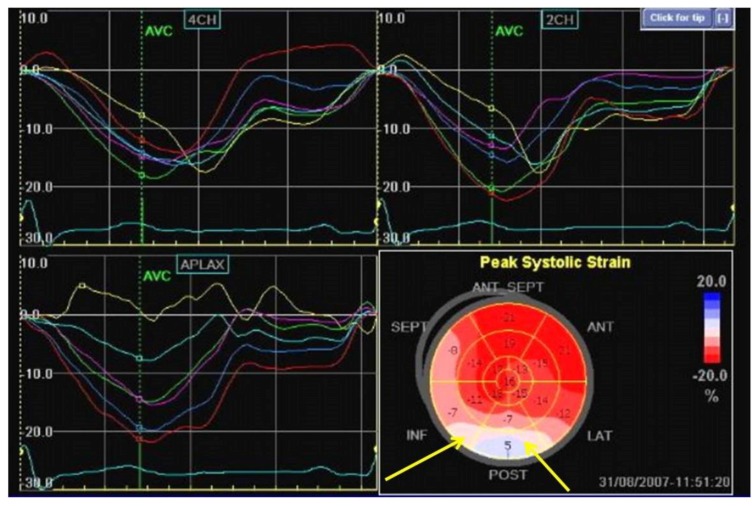
Speckle tracking echocardiography evaluation in patients with previous inferior myocardial infarction notes a reduction of strain value in medium and basal segments of inferior and posterolateral walls (yellow line).

Speckle-tracking echocardiography (STE) allows for the measurement of the different components of active myocardial deformation (strain), providing an indirect evaluation of systolic function and presence of scar/fibrosis [33,34] (Figure 2).

In patients with ischemic dilated cardiomyopathy, a reduction of global longitudinal strain (GLS) has been independently associated with SCD, appropriate ICD therapy, and ventricular arrhythmias [35], also Haugaa et al. have showed, in an observational study including 94 patients with non-ischemic dilated cardiomyopathy [36], that an increase of GLS it is associated with a high risk of arrhythmic events (hazard ratio 1.3; 95% confidence interval 1.1–1.5; *p* = 0.01).

**Figure 3:**
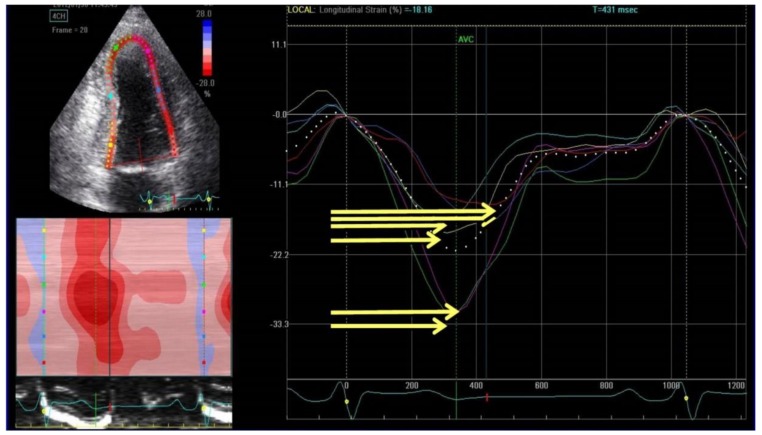
Evaluation of mechanical dispersion by speckle tracking echocardiography. Mechanical dispersion is calculated as the standard deviation of time to peak regional negative strain (yellow lines).

Not only GLS but also mechanical dispersion, defined as the standard deviation of time to peak negative strain from the 16 segments of the left ventricle (Figure 3), was associated to arrhythmic risk in both ischemic and non-ischemic HFrEF [37,38].

In a study of 988 patients with ischemic dilated cardiomyopathy each 10 msec increase of left ventricular mechanical dispersion has been associated with increased the risk of ventricular arrhythmias (hazard ratio 1.24; 95% confidence interval: 1.10 to 1.40; *p* < 0.0004) [39].

From a pathophysiological point of view, these findings are straightforward and highlight that a higher GLS is indicative of a larger scar/more diffuse fibrosis that promotes reentry, and that a large left ventricular mechanical dispersion suggests the presence of the highest anisotropy of the myocardium [40].

However, currently the use of these new echocardiographic markers is only limited to clinical research, and randomized clinical studies are needed for extensive utilization in clinical practice.

### 6.2. Cardiac Magnetic Resonance 

The presence of myocardial scar/fibrosis provides a substrate for malignant ventricular arrhythmias and SCD. Traditionally, assessment of myocardial scar/fibrosis has been based on endomyocardial biopsy, with limitations related to the invasive approach and false negative results (e.g., small myocardial sample size) [41,42], yet today cardiac magnetic resonance (CMR) using the late gadolinium enhancement (LGE) technique can accurately identify and quantify ventricular myocardial scar/fibrosis [43].

A recent meta-analysis assessed the predictive value of CMR late gadolinium enhancement for prediction of ventricular tachyarrhythmia in patients with HFrEF (both ischemic and non-ischemic) dysfunction, in this meta-analysis, 19 prospective studies, with a total of 2850 patients and a mean follow-up of 2.8 years, were identified. The results of these studies demonstrated that LGE is a powerful predictor of ventricular tachyarrhythmias in both groups of patients, and particularly in patients with LVEF 30% or less (odds ratio 5.62; 95% confidence interval: 4.20 to 7.51) [44].

Moreover, Pontone et al. showed that both ischemic and non-ischemic dilated cardiomyopathy patients with positive LGE have a higher rate of major cardiovascular events compared to patients with the same ejection fraction and non-evidence of LGE at CMR (hazard ratio 2.2; 95% confidence interval 1.4 to 3.6; *p* < 0.01) [45].

T1 mapping is the latest method for the quantitative assessment of diffuse fibrosis, providing a reliable quantitative assessment of myocardial tissue [46], recently Chen et al. showed, in a population of 130 patients with HFrEF who underwent ICD implantation, that diffuse interstitial fibrosis assessed with CMR T1 mapping before ICD implantation predicts appropriate ICD discharge during follow-up (hazard ratio 1.10; 95% confidence interval 1.04 to 1.16; *p*< 0.001) [47]. Therefore, these findings support the utilization of CMR for SCD risk stratification, especially in those with borderline systolic dysfunction (i.e., LVEF > 40%) as it can identify patients in which ICD implantation may be beneficial.

### 6.3. Myocardial Sympathetic Innervation Imaging

Sustained activation of the sympathetic nervous system is thought to be a significant contributor to the progression of HF and adverse outcomes including SCD [48]. Myocardial iodine-123metaiodobenzylguanidine (123I-mIBG) imaging provides a non-invasive method to assess cardiac sympathetic function and risk stratify patients with HFrEF [49].

123I-mIBG is a norepinephrine analog, and 123I-mIBG uptake reflects the preservation of cardiac innervation and function of the norepinephrine uptake-1 transporter, reduced 123I-mIBG uptake or accelerated 123I-mIBG washout rate from the heart predicts heart failure progression and death [50] as well being as an indicator of risk of sudden cardiac death and appropriate ICD discharge [51].

Despite these encouraging results, prospective randomized trials will be required to clarify the role of 123I-mIBG in SCD risk stratification in patients with HFrEF.

## 7. ECG and Electrophysiology

### 7.1. Resting ECG

Prolonged interventricular conduction time (i.e., QRS complex duration) promotes ventricular arrhythmias through abnormal dispersion of depolarization and repolarization and resultant cardiac dyssynchrony [52]. Furthermore, in some studies a QRS length >120 msec (irrespective of morphology) predicts SCD in patients with dilated cardiomyopathy, independent of LVEF value [53].

In a subgroup analysis of the MUSTT trial, including 1634 patients with ischemic dilated cardiomyopathy, QRS duration was found to be an independent predictor of overall mortality and SCD in patients with ischemic cardiomyopathy (hazard ratio 1.35; 95% confidence interval 1.08 to 1.69; *p* < 0.001) [54]. However, a meta-analysis including 6138 patients enrolled in eight primary prevention ICD trials [55], failed to prove that QRS duration had an impact on mortality independent of LVEF (hazard ratio 0.78; 95% confidence interval 0.68 to 0.90 vs. hazard ratio 0.70; 95% confidence interval 0.51 to 0.95). 

In a retrospective population-based study (catchment population—one million) a cumulative ECG risk score including heart rate, LV hypertrophy, QRS transition zone, QRS-T angle, corrected QT intervalQTc, and Tpeak-to-Tend was independently associated with SCD (occurred in 522 person) and was particularly useful for a patient with LVEF >35% where risk stratification is currently unavailable (hazard ratio 4.84; 95% confidence interval 2.34 to 9.99; *p* < 0.001) [56].

Despite the above results, prospective studies are needs to clarify the role of resting ECG in SCD risk stratification in HFrEF.

### 7.2. Ambulatory Electrocardiogram 

Historically, 24-hours Holter monitoring was used to predict SCD in patients with HFrEF with ischemic etiology. In fact, patients with previous myocardial infarction and non-sustained ventricular tachycardia (Lown class IV) have higher mortality [57].

However, in one recent multivariate analysis which enrolled 325 patients with myocardial infarction coronary angiography, non-sustained ventricular tachycardia no longer predicted mortality or arrhythmic event (relative risk 1.4; 95% confidence interval 0.3–5.7, *p* = 0.67) [58]. Furthermore, in a recent study which enrolled 319 patients with non-ischemic dilated cardiomyopathy, non-sustained ventricular tachycardia failed to improve SCD risk stratification (hazard ratio 0.93, 95% confidence interval 0.3 to 2.81 *p* = ns) [59].

### 7.3. Autonomic Function Test 

Heart rate variability (HRV) and heart rate turbulence (HRT), have been extensively studied as markers of autonomic dysfunction that have a significant role in the development of ventricular arrhythmias in patients with HFrEF [60].

HRV (i.e., the beat-to-beat variation in either heart rate) is associated with increased ventricular arrhythmias and mortality [61], in the Multicenter Postinfarction Study (MPS), 820 patients were enrolled with acute myocardial infarction and a strong correlation was found between reduced HRV and total mortality following acute myocardial infarction (relative risk 2.7; *p* < 0.0001) [62]. However, HRV does not appear to enhance prognostic stratification when directly compared to other markers of SCD.

Moreover, in patients with non-ischemic cardiomyopathy, HRV is related to left ventricular dysfunction but not to ventricular arrhythmias and SCD [63].

HRT (i.e., the short-term fluctuation in sinus cycle length that follows a ventricular premature complex) is a non-invasive marker of electrical instability that has been shown to identify patients at high risk for SCD [64].

In the non-invasive risk assessment early after myocardial infarction, (REFINE) study enrolled 322 patients HRT was found to reliably highlight patients at a high risk of SCD [65]. However, in non-ischemic population, HRT does not yield predictive power for SCD risk stratification [66,67].

### 7.4. Microvolt T wave Alternans

Microvolt T-wave alternans (MTWA) is a beat-to-beat fluctuation of T-wave amplitude and morphology, that has been associated with increased susceptibility for sustained ventricular arrhythmia [68], early studies showed that TWA was a powerful electrocardiographic tool in predicting SCD in patients, in cohorts with ischemic and non-ischemic cardiomyopathy [69,70].

Despite considerable enthusiasm over TWA, subsequent large prospective trials failed to validate the role of TWA for risk stratification in patients with HFrEF, for example in the Role of Microvolt T-Wave Alternans to Assess Arrhythmia Vulnerability Among Patients with Heart Failure and Systolic Dysfunction (MTWA SCD HeFT) study, in a population of 490 patients, TWA did not predict arrhythmic events or mortality (Hazard ratio 1.24: 95% confidence interval 0.60 to 2.59; *p* = 0.56) [71].

Moreover, the Microvolt T-wave Alternans Testing for Risk Stratification of post-MI patients (MASTER) study, that enrolled 575 patients with previous myocardial infarction, showed that MTWA did not predict a composite endpoint of arrhythmic death and appropriate ICD discharge (hazard ratio: 1.26; 95% confidence interval: 0.76 to 2.09; *p* = 0.37) [72].

### 7.5. Signal-Averaged Electrocardiography

**Figure 4:**
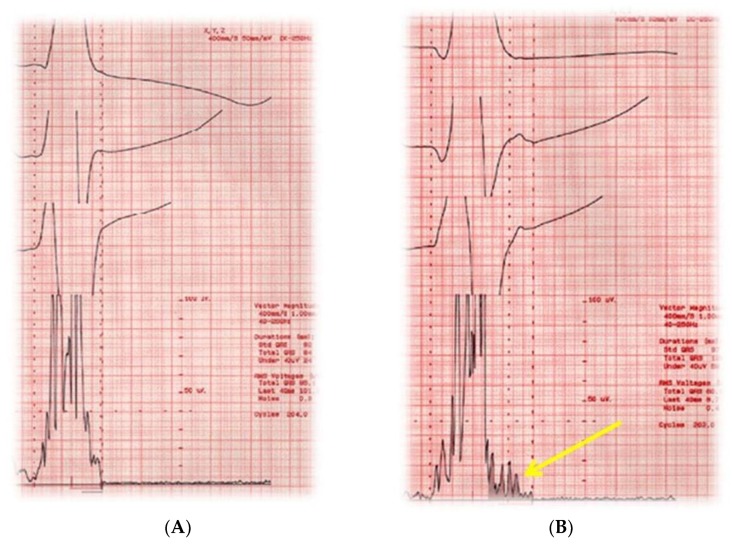
Signal-average ECG of a normal subject (**A**) and a patient with idiopathic dilated cardiomyopathy (**B**). Note the fragmentation of terminal parts of QRS indicative of late potentials (yellow line).

Signal-averaged electrocardiography (SAECG) is a method to improve the signal-to-noise ratio of a surface electrocardiogram, thus facilitating the identification of low-amplitude signal at the end of QRS complex (Figure 4) [73].

This test was initially found to be a promising marker of SCD in patients with ischemic cardiomyopathy, and later in patients with non-ischemic cardiomyopathy [74]. Nevertheless, subsequent studies have shown conflicting and less promising results. A more recent study performed, comparing 123-mIBG to SAECG, showed that 123-mIGB, and not SAECG, was a predictor of SCD in patients with mild to moderate systolic dysfunction [51].

However, SAECG has a high negative predictive value (normal SAECG is associated at risk <5% on inducible ventricular tachycardia at the electrophysiology study). For this reason, SAECG may be used as the first-line test to identify patients at low risk of SCD.

### 7.6. Electrophysiologic Study

Early studies on the use of invasive electrophysiology (EP) aiming to study risk-stratify patients at increased risk of SCD in ischemic HFrEF showed conflicting results, with nearly half of all studies finding that the inducibility of sustained ventricular tachycardia (VT) was unhelpful in predicting later mortality or arrhythmic events [75].

A sub-study of the Multicenter Automatic Defibrillator Implantation Trial II (MADIT II) trial showed that inducible ventricular tachycardia was inversely related with ventricular fibrillation requiring defibrillation [76].

In patients with non-ischemic dilated cardiomyopathy, EPS shows many limits in the risk stratification of SCD related to low inducibility of ventricular tachycardia [77] and low predictive positive value of induced tachycardia for SCD risk stratification [78].

In conclusion, the utility of EPS may be confined to HFrEF of ischemic etiology and may lie in its combined use with other non-invasive tests, such as MTWA and HRV, to further refine the selection of potential ICD recipients [79].

## 8. Biomarkers 

Since HF is a complex clinical syndrome, a single biomarker might not reflect all of its characteristics. For this reason, a combined biomarker approach is required for accurate clinical decision-making.

Natriuretic peptides (NPs) play an essential role in the diagnosis and prognostic stratification of HFrEF [80]. In a study including 521 patients with previous myocardial infarction, brain natriuretic peptides (BNP) provided information of the risk of subsequent SCD independent of other clinical variables and left ventricular ejection fraction (relative risk 3.39; 95% confidence interval 1.22 to 9.45; *p* = 0.037). In another study, 398 patients with HFrEF were enrolled and BNP increase was associated with an increased risk of SCD only in patients with QTc interval prolongation (hazard ratio 1.63; 95% confidence interval 0.54 to 5.12; *p* < 0.001) [81]. NPs may have a role in SCD risk stratification in HFrEF, however the variation of their levels, due to systemic congestion or the presence of caveats to their interpretation, must be considered in clinical practice. 

Many reports have suggested that an increase in serum cardiac troponin (cTnT) or cardiac troponin I concentrations is a reliable indicator of myocyte injury in patients with HF, leaving aside the presence of myocardial ischemia. Recently, Nakamura et al. found, in a population of 70 patients with stable HFrEF [82], that TnT levels were associated with SCD (Hazard ratio 10.5;95% confidence interval 2.97 to 48.7 *p* < 0.001). Soluble ST2 (sST2), a member of the interleukin-1 family, is a novel biomarker that reflects cardiovascular stress and fibrosis with potential prognostic implications in patients with HFrEF. In a case-control study including 32 cases of SCD from MUSIC (MUerte Súbita en Insuficiencia Cardíaca) registry, sST2 was a strong predictor of SCD (odds ratio 4.56, 95% confidence interval 1.31 to 15.9, *p* = 0.017) [83]. Despite the above results, prospective studies are needs to clarify the role of biomarkers in SCD risk stratification in HFrEF. 

## 9. Genetics and Genomics

Multiple population-based studies exist suggesting a substantial genetic contribution to individual SCD risk, independent of LVEF [84], for example The Seattle case-controlled study demonstrated an increased risk of SCD among patients with a positive family history or parental history of early-onset SCD [85].

Despite the multiple studies demonstrating a strong association between family history and SCD [86], there has yet to be a specific genetic variant or clinical marker identified that has proven effective in predicting individual risk. Furthermore, due to the variability in the mechanism of SCD, it is likely that there is a broad spectrum of heritability of SCD in different populations [87].

For example, in inherited dilated cardiomyopathy lamin, A/C and desmin mutation carry an increased risk of SCD [88,89,90].

Recently, genome-wide association studies (GWAS) have been performed to isolate genetic variants modulating SCD risk, with a specific interest in genes that play a role in structural abnormalities [91]. 

However, these initial observations are limited in clinical applicability at this time due to the small sample size.

## 10. Artificial intelligence and machine learning

Artificial intelligence (AI) is rapidly simplifying all aspects of human life. Machine learning (ML), allows machines to acquire information by extracting models from large databases and is increasingly used in the field of cardiovascular diseases. In recent years, ML has been used to facilitate the diagnosis and prediction of adverse events in patients with HFrEF. Regarding the stratification of the risk of sudden cardiac death, significant steps have been achieved in recent years, for example, Manis et al. have shown that the use of ML applied to HRV analysis allows stratifying HFrEF patients at high or low risk of SCD [92]. Studies regarding the use of ML applied to cardiac imaging to improve SCD risk stratification in HFrEF are ongoing [93].

## 11. Risk stratification of SCD in HFpEF

In the recent HFpEF trials, SCD was reported to be one of the leading causes of death. In the CHARM (Candesartan in Heart Failure-Assessment of Reduction in Mortality and Morbidity) trial the SCD represented 28% death for all causes [94]. Similarly, 26% of all deaths in the I-PRESERVE (Irbesartan in Patients with Heart Failure and Preserved Ejection Fraction) trial were due to SCD [95].

In the ongoing KaRen (Karolinska Rennes) study, including 539 patients with HFpEF, many clinical and echocardiographic parameters (atrial fibrillation, left atrial volume index, E/e’ ratio, and pulmonary pressure) were found to be related to clinical outcome. However, none of these parameters were specific for SCD [96,97,98]. Instead, recent data from the TOPCAT (Treatment of Preserved Cardiac Function Heart Failure with an Aldosterone Antagonist trial) male sex (hazard ratio 2.12; 95% confidence interval 1.27 to 3.53), non-white race (hazard ratio 3.37; 95% confidence interval 1.53 to 7.44), and insulin-treated diabetes mellitus (hazard ratio 2.55; 95% confidence interval 1.54 to 4.22) identify patients at high risk for SCD with modest discrimination (C statistic 0.68) [14].

In clinical practice, evaluation of SCD risk in patients with HFpEF is particularly challenging both for the extreme phenotypic variability of patients and for the high percentage of non-cardiovascular death. In Table 2 the recognized risk factors for in HFpEF patients are reported.

## 12. Conclusions

**Figure 5:**
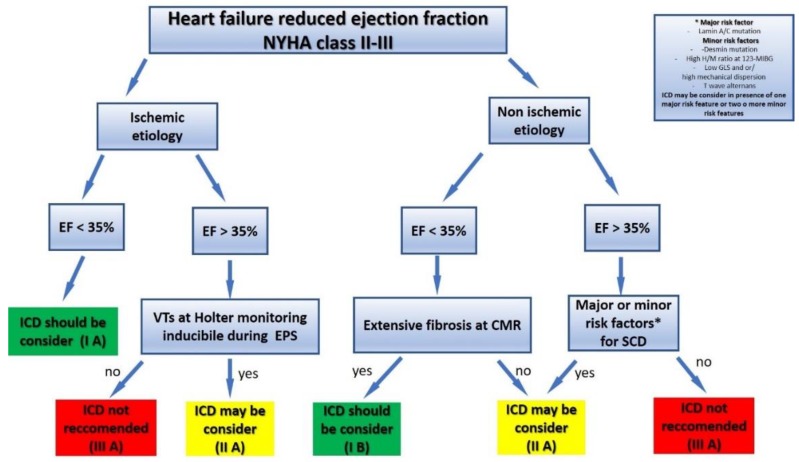
Proposed risk stratification of sudden cardiac death in patients with heart failure reduced ejection fraction. EF: Ejection fraction, VTs: Sustained ventricular tachycardias, ICD: Implantable cardioverter defibrillator, CMR: Cardiac magnetic resonance, H/M ratio: Heart /mediastinum ratio, GLS: Global longitudinal strain.

Despite progress in pharmacological and electrical therapy, SCD remains the most frequent cause of mortality in patients with HFrEF. Early studies have identified LVEF and ischemic etiology as predictors of events. However, the epidemiological change in SCD confirmed an unmet need for risk stratification. In this regard, the most recent imaging, advanced electrocardiographic techniques, and genetic tests can enhance the identification of high-risk individuals who may benefit from a specific approach to prevent SCD (Figure 5).

On the other hand, HFpEF patients also have a high prevalence of SCD. For this patient, a better understanding of pathophysiologic patterns of SCD may improve our risk stratification of this entity.

## Figures and Tables

**Table 1:** Risk factor for SCD in HFrEF.

**Familial and Personal History**
-Prior cardiac arrest
-Family history of SCD
-Unexplained syncope
**Electrocardiography**
-QRS duration
-T wave alternans
-Signal averaged ECG
**Autonomic Function**
-Heart rate variability
-Heart rate turbulence
**Electrophysiologic Study**
-Inducible ventricular arrhythmias
-Extensive low voltage/abnormal signals on electroanatomic mapping
-Large mid-epicardial scar burden
-Multiple VT morphology
**Echocardiography**
-LVEF
-Ventricular dyssynchrony
-Speckle tracking
-Mechanical dispersion
**Cardiac MRI**
-Late gadolinium enhancement
-T1 mapping
**Myocardial Sympathetic Innervation Imaging**
-Heart to mediastal ratio
**Biomarkers**
-Natriuretic peptides
-High sensitive troponin
-Soluble ST2
**Genetics**
-Lamin A/C mutation
-Desmin mutation

SCD, sudden cardiac death; VT, ventricular tachycardia; LVEF, left ventricular ejection fraction; QRS: QRS complex.

**Table 2:** Risk factors for SCD in HFpEF.

**Personal History**
Age
Male sex
Insulin-treated diabetes mellitus
Prior myocardial infarction
**Electrocardiogram**
Left bundle branch block
**Biomarkers**
Natriuretic peptides
